# Free-Living Amoebae as Hosts for and Vectors of Intracellular Microorganisms with Public Health Significance

**DOI:** 10.3390/v9040065

**Published:** 2017-04-01

**Authors:** Carsten Balczun, Patrick L. Scheid

**Affiliations:** 1 Laboratory of Medical Parasitology, Central Institute of the Bundeswehr Medical Service, Koblenz, Andernacherstrasse 100, 56070 Koblenz, Germany; carsten.balczun@rub.de; 2 Institute of Integrated Sciences, Department of Biology, Parasitology and Infection Biology Group, University of Koblenz-Landau, Universitätsstrasse 1, 56070 Koblenz, Germany

**Keywords:** free-living amoebae, FLA, *Acanthamoeba*, vectors, hosts, endocytobionts, endosymbionts, *Pandoravirus*, *Mimivirus*, *Pithovirus*, public health

## Abstract

Free-living amoebae (FLA) are parasites within both humans and animals causing a wide range of symptoms and act as hosts of, and vehicles for phylogenetically diverse microorganisms, called endocytobionts. The interaction of the FLA with sympatric microorganisms leads to an exceptional diversity within FLA. Some of these bacteria, viruses, and even eukaryotes, can live and replicate intracellularly within the FLA. This relationship provides protection to the microorganisms from external interventions and a dispersal mechanism across various habitats. Among those intracellularly-replicating or -residing organisms there are obligate and facultative pathogenic microorganisms affecting the health of humans or animals and are therefore of interest to Public Health Authorities. Mimiviruses, Pandoraviruses, and Pithoviruses are examples for interesting viral endocytobionts within FLA. Future research is expected to reveal further endocytobionts within free-living amoebae and other protozoa through co-cultivation studies, genomic, transcriptomic, and proteomic analyses.

## 1. Introduction

Free-living amoeba (FLA) are protozoa ubiquitously found in nature. They form a rather heterogeneous group of facultative parasitic amoebae within the free-living protozoa without common phylogenetic, systematic, or taxonomic origin [1]. Among the FLA, only four genera are pathogenic for humans and animals: *Acanthamoeba*, *Balamuthia*, *Naegleria*, and *Sappinia*. The taxonomy of FLA was revised several times in consequence of the availability of genomic sequence data. Regarding morphologic, biochemical and molecular data, a new taxonomic classification was proposed by the International Society of Protozoologists. According to this classification these pathogens has been placed in two ‘Super Groups’. *Acanthamoeba* and *Balamuthia* were classified under the super group Amoebozoa: *Acanthamoebidae*; *Naegleria fowleri* under the super group Excavata: Heterolobosia: *Vahlkampfiidae*; and *Sappinia* under the super group Amoebozoa: Flabellinea: *Thecamoebidae* [2].

The FLA are widespread in many habitats including soil, dust, air, seawater, drinking water, swimming pools, sewage, eyewash solutions, contact lenses, dialysis units, and dental treatment units [3]. Their prevalence in water networks is associated with biofilms, where they live sympatric within a biocoenosis together with other microorganisms. These biofilms serve as feeding grounds for the FLA and provide protection to a certain degree while the FLA adhere to their surfaces. They play a role in the reduction of bacterial biomass and the regeneration of nutrients. In such a biocoenosis there are multiple interactions between FLA and other microorganisms.

The trophozoites of FLA are the vegetative form and in this state, they can serve as prey for other microorganisms, such as amoebophagous fungi [4] or act as aerobic heterotrophic predators by feeding on algae, bacteria, cyanobacteria, and fungi, as well as on smaller protozoa. However, there are numerous pathogenic microorganisms of hygienic relevance which may survive and evade phagocytosis and digestion within the FLA trophozoites. One protective mechanism used by some species is the cyst. This life stage protects the organism from adverse environmental conditions and, therefore, makes these pathogenic organisms enormously resistant, persistent, and tenacious.

This review highlights the pathogenic potential of certain FLA and the consequences for public health caused by their interactions with other microorganisms.

## 2. Pathogenic Free-Living Amoebae

For a long time, FLA were considered to be harmless protozoa of soil and water. However, research since the 1960s has demonstrated that FLA can be pathogenic to humans and animals [5] with near 100% morbidity from some strains. When FLA are in intimate contact with humans they can provoke serious infections, for example by using contaminated contact lenses. The following section describes the diseases caused by the four well-characterized pathogenic genera of FLA, *Acanthamoeba*, *Balamuthia*, *Naegleria*, and *Sappinia*, as well as genera most recently recognized to be involved in infections, and even newly-described species.

The *Acanthamoebae* are the most well-known facultative parasitic FLA. Their pathogenicity varies among their species or strains which are currently divided into 18 sequence types (genotypes; T1–18) based on the nuclear small-subunit ribosomal RNA (rRNA) gene, rather than their morphology. The predominant genotype in human pathogenic *Acanthamoebae* is T4; other genotypes have also been reported as pathogens [6,7,8,9]. As etiological agents of the so-called acanthamoebiasis, they can trigger several specific disease symptoms in humans. The amoebiasis of the central nervous system is called granulomatous amebic encephalitis (GAE). The infection route of *Acanthamoebae* mostly include the lower respiratory tract and skin lesions followed by hematogenous spread, finally reaching the central nervous system through the blood-brain barrier [3]. GAE differs clinically and morphologically from the primary amoebic meningoencephalitis (PAM), which is caused by *Naegleria fowleri*, showing subacute to chronic progress [10,11]. To date, approximately 150 cases have been reported worldwide and the infection is almost always fatal because of the difficulty and delay in diagnosis and lack of optimal therapy [3].

Cutaneous acanthamoebiasis is a rare opportunistic infection and occurs almost exclusively in immunocompromised persons, similar to GAE. Characteristic lesions of the skin, such as ulcers and erythematous sores, contain *Acanthamoeba* trophozoites and cysts and are a route of entry into the blood stream, with subsequent hematogenous spread to different tissues. Alternatively, the *Acanthamoeba* keratitis is not necessarily associated with an immune suppression, but rather with a trauma, exposure to contaminated water or, particularly, the improper handling of contact lenses, which promotes infection [12,13].

The *Naegleria* infection is a rare disease, which is caused by *Naegleria fowleri*. Up to now, only *Naegleria fowleri* is known in humans as an etiological agent of PAM, which has a worldwide distribution of more than 300 reported cases, mostly from the United States [14]. It is almost always fatal; only three people in the United States out of 138 have survived infection [15]. This infection generally occurs in previously healthy children and young adults with a history of swimming and other recreational activities in warm freshwater lakes and ponds [16,17]. When contaminated water enters the nose *N. fowleri* reaches the brain by migration alongside the olfactory nerves [18]. The naegleriosis shows up as a diffuse meningoencephalitis with swollen and edematous cerebral hemispheres. After a critical period with ataxia, convulsions, and coma, the patient may die within a very short period of time [16,17].

The clinical picture of GAE by *Balamuthia mandrillaris* is characterized by headache and neck stiffness. The *Balamuthia* infection is also known as granulomatous *Balamuthia* encephalitis (BAE) [19]. So far, approximately 100 cases have been reported, and only three survivors are known. The infection is chronic and the time between infection and appearance of neurological symptoms may range from one month to about two years [3]. The portal of entry are mostly skin lesions contaminated by soil and most cases of BAE are reported from warm regions [20]. However, quite recently, DNA of *B. mandrillaris* was found unexpectedly in a routine screen for *Acanthamoeba* in a contact lens sample. This demonstrates how easily pathogens might be missed if their occurrence is not expected [21].

The two-nucleated *Sappiniae* have been identified only once as an etiological agent of an amoeba-associated encephalitis with a favorable clinical outcome [22]. This amoebic encephalitis (SAE) was first associated with *Sappinia diploidea*, while an affiliation to *Sappinia pedata* was later proved using molecular biological studies [23].

*Hartmannella* sp. has also been discovered in keratitis cases, but determining whether they were pathogens or merely contaminants, still remains controversial [24,25]. The same applies to Vahlkampfiae, which were found together with Hartmannellae while diagnosing a keratitis of a contact lenses wearer [26]. *Paravahlkampfia francinae*, a recently described species within the genus *Paravahlkampfia*, was isolated from the cerebrospinal fluid of a patient with PAM-like symptoms, usually caused by *Naegleria fowleri* [27]. *Allovahlkampfia spelae*, a newly-discovered amoeba, was identified as a cause of chronic human keratitis in an Egyptian patient in 2016 [28]. The only involvement of *Dictyostelium polycephalum*, a social amoeba, was described in 2010 in a human keratitis in India [29]. The infection occurred in an immunocompetent person without history of ocular injury or surgery.

## 3. FLA as Vectors of Diverse Microorganisms (Biodiversity within FLA)

Biofilms serve the FLA as basic food and protection sources, where the FLA adhere as trophozoites on the surfaces [30], and they interact with many different microorganisms in their biocoenoses, especially in these biofilms. While the FLA usually use bacteria and other microorganisms as food sources, some of the prey organisms manage to survive within their grazing predators or even proliferate within their cytoplasm or their nucleus.

The generic term ’endocytobionts’ was introduced because the interaction of FLA with phylogenetically diverse microorganisms lead to a plethora of different outcomes [5,12,31]. This term avoids any predetermination of possible features of these ’relationships’, such as parasitism, symbiosis (‘endosymbionts’), phoresy, zoochory, or mutualism, especially since the behavior of the endocytobionts may vary depending on the temperature (see *Legionella*) or other conditions [5].

FLA play an important role in the transmission and dispersal of pathogenic mircroorganisms by acting as a ‘trojan horse’ serving as vehicles for the colonization of new habitats or hosts. The relationships between FLA and the diverse intracellular microorganisms shows similarities to the relationships between some prokaryonts and macrophages: the virulence of pathogenic microorganisms to their amoebal host indicates a virulence and an adaptation towards human or animal cells, e.g., macrophages [32,33,34,35,36]. FLA serves as a ‘training ground’, a prerequisite for a predation-induced adaptation to life in a eukaryotic host [5,37,38,39,40]. It is known that the virulence in bacteria is often correlated to resistance to antibiotics or disinfection measures [41,42]. Conclusively, FLA-endocytobiont relationships are of relevance for the origin of pathogens and they make an impact on evolutionary processes [5]. The impact of the host-endocytobiont relationship may, in turn, also lead to an increase of virulence of the FLA [5,41,43].

## 4. Host-Microorganism Interactions

### 4.1. Amoebal Co-Culture vs. In Situ Interactions

The analysis of interactions of FLA and pathogenic bacteria species, or other microorganisms, are of great value for public health. Since mechanisms allowing bacteria to escape from phagocytosis and digestion by FLA are believed to be similar to the mechanisms allowing the same microorganisms to evade a similar fate by macrophages, co-culture with FLA has been proposed as a new tool to recover and identify potentially new pathogens from environmental samples [44]. By incubating samples on axenically-grown FLA, and subsequent staining and visualization of microorganisms, the putative development of bacteria inside FLA can be analyzed. Molecular methods, like sequencing of 16S ribosomal DNA (rDNA), are subsequently used to identify bacterial isolates [45]. The careful selection of specific FLA for these experiments is necessary since the host range of pathogens might be narrow and, therefore, by choosing the wrong (those not susceptible or insensitive) FLA-strain, the isolation of a specific pathogen might be missed. Expanding the panel of used FLA or other protozoa is, therefore, promising [46].

A restriction of this widely-used co-culture experiments is the in vitro framework of the laboratory assays. Although the chosen FLA and the microorganism might be inhabitants of the same natural habitat, the interaction is exclusively shown for the precise experimental conditions. Hence, chosen protocols for the recovery of intra-amoebic microorganisms might miss the particular condition required for a specific interaction (e.g., bacteria/amoeba ratio or temperature). There is a significant qualitative difference, as to whether the microorganisms could be isolated from a real specimen (e.g., an environmental sample or patient specimen) or solitary under laboratory conditions. It is another qualitative differentiation mark, whether the endocytobionts are only transported by FLA as vector/carriers, or whether they are able to proliferate intracellularly with the FLA as the host [5]. Table 1 gives an overview of a selection of microorganisms of public health significance interacting with FLA in which in situ and in vitro interactions are listed separately. A comprehensive list of pathogens, including the nature of interaction between bacterial pathogen and amoebal host, was published by Thomas et al. [44].

### 4.2. Interactions of FLA and Bacteria

Among the numerous bacterial species interacting successfully with FLA, or even other protozoa, many are known to be pathogenic for humans [118]. More than 100 proven pathogens for humans are described as surviving and/or proliferating when in contact with various amoebal species [44]. These impressive numbers seem to be underestimated since in most studies analyzing host-pathogen interactions often just one, typically *A. polyphaga*, or just a few protozoa species/strains were used and a range of bacterial pathogens have not yet been tested to interact with FLA. Important pathogens like mycobacteria, *Legionella pneumophila*, *Legionella micdadei*, *Vibrio cholera*, and *Francisella tularensis* are able to survive not only in trophozoites but even in cysts [100,101,119,120,121,122,123]. Since bacteria incorporated in trophozoites show increased resistance to biocides, the resistance of bacteria inside cysts is even more increased [74,121,124]. Hence, pathogenic bacteria internalized in amoebae represent a high risk for public health because they are not reached by conventional biocide treatment. Some of these bacteria-FLA interactions are described below in more detail.

#### 4.2.1. *Legionella pneumophilia*

*Legionella pneumophilia* was first described more than 35 years ago, as the etiological agent of the Legionaries’ disease. It is widespread in nature and can proliferate inside FLA like *Acanthamoeba* spp., *Naegleria* sp., *Hartmannella* sp. and other protozoa [5]. Legionella is able to avoid degradation by the phagolysosomes in the amoeba and hence, FLA might also contribute to the virulence characteristics of the bacteria interacting with human macrophages [125].

Co-cultivation with FLA leads to regeneration of bacteria from the so called ’viable-but-non culturable’ state due to starvation or biocide application as was shown for *Legionella pneumophilia* [126]. Beside this resuscitation effect of amoebae on *Legionella*, the interaction also enables the bacterium to tolerate elevated concentrations of biocides. Only a 10-fold kill was achieved with an isothiazolone derivate against intra-amoebal-grown *L. pneumophilia* whereas, for in vitro-grown bacteria, a 1000-fold kill was observed [127]. Thus, insufficient biocide application might generate serious problems since internalization makes pathogens motile with the FLA as vectors or transport vehicles for the pathogenic bacteria.

#### 4.2.2. FLA-Bacteria Interactions Other than Legionella

The facultative intracellular pathogen *Listeria monocytogenes* is a food-borne opportunistic pathogen which can switch from an environmental saprophyte to a potentially fatal human pathogen. *L. monocytogenes* is not digested when incorporated in *Acanthamoeba* and bacteria are released from vegetative trophozoites, whereas bacteria are killed in cysts [56].

*Campylobacter jejuni* is able to resist digestion in *Acanthamoeba* for a long time and cells survived for longer periods when co-cultured with *Acanthamoeba* sp. than when grown in culture alone [75,128]. In addition, moderately acidic conditions were shown to trigger *Campylobacter jejuni* adhesion and internalization into *Acanthamoeba* polyphaga [129]. Therefore, improper biocide application might increase internalization of pathogens. Co-cultivation of *C. jejuni* with *A. castellanii* increases its survival of chemical disinfection with an iodine-containing product [75]. Additionally, transmission of endocytobionts has been reported (e.g., *Campylobacter jejuni* in *Acanthamoeba castellanii* to broilers) [130].

Within the *Mycobacterium* genus, the species *Mycobacterium tuberculosis* and *Mycobacterium leprae* are considered to be the most important human pathogens. *Mycobacterium avium* and other species of the *Mycobacterium* genus are opportunistic pathogens having a high impact for HIV/AIDS patients [131]. *Mycobacterium avium* enters and replicates in *A. castellanii* and amoeba-grown mycobacteria have an enhanced virulence in the mouse model [41].

*Arcobacter butzleri* can establish a long-lasting relation with *Acanthamoeba castellanii* for up to 10 days in laboratory co-culture experiments [132]. The pathogen is also able to replicate inside the amoeba suggesting a possible endocytobiotic interaction and a possible role of the FLA as a transport vehicle for the bacteria [76,77]. In co-culture experiments with *Acanthamoeba castellanii*, *Yersinia enterocolitica* can evade digestion and survives at least 14 days in nutrient-poor and nutrient-rich medium. The bacteria seem to evade from digestive vacuoles and replicate within the cytoplasm of the amoeba [133]. Ingested *Yersinia* were protected from chlorination since the pathogen has increased resistance to free chlorine residues [74]. Chlorine is a widely used chemical agent for disinfection of surfaces and, therefore, a failure of disinfection due to internalization of *Yersinia* by FLA has to be considered.

*Salmonella typhimurium* replicates inside contractile vacuoles and even food vacuoles of *Acanthamoeba polyphaga* to a high degree (100–200 cells per vacuole) [72]. Due to the high density of cell growth, the protozoa is now considered to be an environmental reservoir for this bacteria [78]. The interaction of *S. typhimurium* and *Salmonella dublin* with *Acanthamoeba polyphaga* and *Acanthamoeba rhysodes* upregulates pathogenicity factors of the bacteria indicating an enhancement of virulence through the amoebal passage [73,134]. In addition, exposure of *Salmonella typhimurium* to different rumen protozoa also enhances virulence of the bacterium indicating that virulence-enhancement is a common mechanism in this relationship [135,136,137].

## 5. Viruses in Close Association with FLA

Viruses are highly abundant and ubiquitous components of the biosphere. Their genomes are extremely variable concerning their composition, organization and size [138]. Thus, viruses represent specialized, simple organisms that interact in their intracellular environment with potential hosts and sympatric occurring microorganisms [139]. The story of viruses in close association with FLA began in 1974 when Schuster and Dunnebacke detected virus-like particles in *Naegleria* sp. [140].

### 5.1. Coxsackievirus and Adenovirus

The human pathogenic coxsackievirus B3 (CVB-3) is incorporated in *Acanthamoeba* trophozoites without changing the infective activity of the virion [112]. In co-culture experiments CVB-3 remains infectious even after a complete 6-month cycle of encystment and excystment. *Acanthamoeba* are therefore suggested to play an active role as a vector in the survival of coxsackieviruses. *Acanthamoebae* are also considered to be a potential reservoir and a vector/carrier of adenoviruses to humans [111]. Viral DNA of different human adenoviruses (HAdv) serotypes has been found in *Acanthamoeba* isolates from water sources in the Canary Islands [108]. The serotype HAdV-2 was found to be the most frequently detected.

### 5.2. Mimiviridae

Additionally, the interaction of these proven human pathogenic viruses with amoebae the discovery of the so-called giant viruses has increased interest in the association of viruses and FLA. The 0.8 µm large *Acanthamoeba polyphaga Mimivirus* (APMV) was described in 2003 and placed in the family of the *Mimiviridae* [109]. It was isolated in 1992 (from a cooling tower in the UK at Bradford) and this intracellular endocytobiont developing within *Acanthamoebae* was first considered as a bacterium which was finally named ‘Bradford coccus’ [109]. This misinterpretation still reflects in the naming of Mimiviruses (‘mimicking Microbe virus’). Virion factories were described as functional dynamic structures, modifying areas of the infected trophozoites to assure viral morphogenesis [141]. To date, about 100 amoebal Mimiviruses from various environmental water and soil samples, but also human samples have been isolated [142]. Phylogenomics separate these viruses in three lineages named A, B, C with their main representatives *Mimivirus*, *Acanthamoeba polyphaga moumouvirus*, and *Megavirus chilensis*, respectively. *Megavirus chilensis* was isolated from a marine sediment sample in the aftermath, which had even a larger genome than the Mimivirus. Already at this time, it was evident that the extent of the complexity of these viruses that multiply in protozoa, is not yet able to be predicted.

### 5.3. Marseilleviridae

About five years after the discovery of the *Acanthamoeba polyphaga Mimivirus*, the *Acanthamoeba polyphaga Marseille* virus was isolated from a cooling tower in Paris and placed taxonomically within the *Iridoviridae* family [143]. This giant virus belonged to a further new family within the nucleocytoplasmatic large DNA viruses (NCLDV) and showed at that time the fifth-largest virus genome. A separate order (Megavirales) was proposed to combine the giant viruses such as the *Marseillevirus* and related viruses [142,144]. Since the discovery of the giant virus in amoebae it has been searched extensively (especially in biofilms and sediments) for other viruses as endocytobionts in FLA and many new representatives have been found [142]. It could never be excluded that the Megavirales could actually represent a separate branch of micro-organisms different from previously described domains of life and, thus, initiate a revolution in the classification of micro-organisms.

### 5.4. Pandoraviruses and Pithoviruses

**Figure 1:**
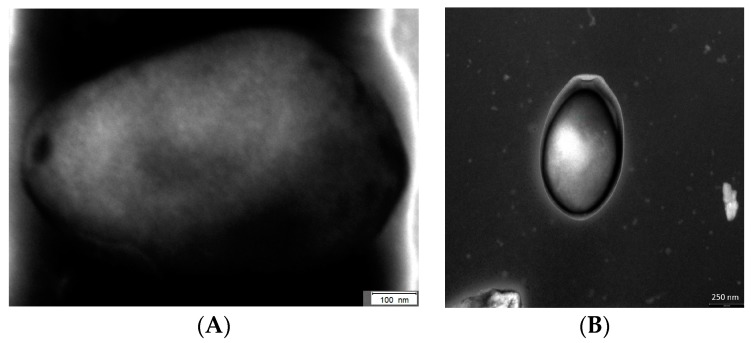
Transmission electron microscopic picture of *Pandoravirus inopinatum* by negative staining contrast. The bar in (**A**): 100 nm, and the bar in (**B**): 250 nm (staining performed by Dimitrios Frangoulidis and Claudia Kahlhofer; Bundeswehr Institute of Microbiology, Munich).

About 10 years after the discovery of the *Mimivirus* in *Acanthamoebae*, more giant viruses in FLA were investigated and named as Pandoraviruses. It turned out that in 2008 the first *Pandoravirus* has been described in its natural host while the taxonomic affiliation was not solved at that time [12,115,145]. These viruses do not have any similarity with the presently-known virus families on the genomic level. Even morphologically the virion was not comparable to any other viral structure (Figure 1) [146]. So far, three species of these extraordinary viruses are analyzed in detail, *Pandoravirus salinus*, *Pandoravirus dulcis*, and *Pandoravirus inopinatum* [115,147,148]. Virion factories have never been clearly observed in Pandoraviruses like in *Mimivirus*. However, similar structures have been proposed for the replication of Pandoraviruses [146,149].

Pithoviruses were analyzed on the genomic level in 2014 when a sample from >30,000-year-old Siberian permafrost was inoculated on *Acanthamoeba castellanii* host amoeba [116]. Similar to *Pandoravirus*, a relative of Pithoviruses, the endocytobiont KC5/2 was first described by Hofmann et al. before the identity as a giant virus was evident [110]. The *Pithovirus sibericum* particle/virion is 1.5 µm in length but has a smaller genome compared with the Pandoraviruses [116,150].

### 5.5. Faustovirus and Mollivirus

Several isolates of Faustovirus, an Asfarvirus-related lineage of giant viruses, were isolated using *Vermamoeba vermiformis*, the first time an FLA other than *Acanthamoeba* sp. has been used [46]. This virus forms icosahedral virions of 200 nm and the genome encodes for 451 predicted proteins from which about two-thirds do not show significant similarity to known protein-encoding genes. Prominent virus factories were built within the host cells and complete viral particles were released at 18 to 20 h post-infection [46].

*Mollivirus sibericum* was isolated from the same permafrost sample as *Pithovirus sibericum* [117]. The spherical virion of 0.6 µm diameter differs significantly from *P. sibericum* and encloses a genome which encodes 523 proteins, 16% of them have their closest homologies to proteins from *Pandoravirus*. Astonishing 10% of the encoded proteins may have been acquired by horizontal gene transfer from its host since their closest homologs are from *Acanthamoeba castellani* [117].

### 5.6. Giant Viruses as Putative Pathogens

Most of the viruses associated with FLA are isolated by using amoebal co-cultivation. Therefore, except for APMV and *Pandoravirus inopinatum*, which were isolated together with their respective *Acanthamoeba* host, and *Pandoravirus inopinatum* even from a patient specimen, the real host in the environment is not known [12]. Although their potential pathogenicity for humans is not clear, at least for *Pandoravirus inopinatum*, it was shown how easily theses giant viruses get in close contact to humans. In addition, APMV interacts with the interferon system of vertebrates and replicate in human and murine phagocytes [151]. Serology studies support the role of mimiviruses in hospital-acquired pneumonia [152]. Hence, the possible role of these giant viruses as pathogen needs to be clarified.

### 5.7. Giant Viruses and Evolution

The discovery of these large DNA viruses led to questioning the evolutionary significance of these unusual viruses, because viruses are generally in much closer relationship to their host cells as to other viruses [153]. Features of complex genetic interaction such as interviral gene transfer, the correlation to virophages (e.g., Sputnik), lateral gene transfer, and their role with respect to their evolution, remained enigmatic for a long time. It was known that the laterally acquired genes are associated with the sympatric lifestyle (in association with viruses, bacteria, and eukaryotes) and the simultaneous occurrence of virophages [154,155]. Lateral gene transfer obviously occurred to a great extent in *Acanthamoeba castellanii* and led to integration of viral genes from all known giant NCLDVs [156]. Some of these genes are actively transcribed in the amoeba suggesting their domestication during amoebal evolution. Giant viruses, such as the *Marseillevirus* use their amoebal hosts (e.g., *Acanthamoebae*) as ‘gene melting pots’, a place where horizontal gene transfer occurs. The FLA play a role in the genesis of complex, chimeric genomes [113], for example *Mimivirus* acquired tyrosyl-tRNA synthetase from the host amoeba *Acanthamoeba polyphaga* [155].

Most recently, a comprehensive analysis of the nuclear genome of the *A. castellanii* Neff strain suggests the existence of a yet-undiscovered family of amoeba-infecting NCLDV [156]. A bioinformatics tool which especially screens metagenomes for giant virus-related sequences might improve our knowledge about the presence and prevalence of giant viruses in the environment and the human body [157]. Therefore, the story of giant viruses in amoebae and the discovery of new viruses seem to be going on.

## 6. Interactions of FLA with Protozoa or Fungi

Only a few examples of interaction between FLA and protozoa (and also fungi) have been analyzed so far, including examples of endozoochory or parasitism [61,105]. *Cryptosporidium parvum* causes cryptosporidiosis, a parasitic disease of the mammalian intestinal tract [106]. Especially immunocompromised individuals, including HIV/AIDS patients, often suffer from intractable diarrhea, which can be fatal. In co-cultivation experiments the capacity of *Acanthamoeba* to predate *Cryptosporidium* oocysts was demonstrated [105,107]. Winiecka-Krusnell et al. examined the interaction of FLA and *Toxoplasma gondii* [158]. Oocysts were actively internalized and can establish infection in a murine model.

*Cryptococcus neoformans* is a microscopic fungus that lives in the environment throughout the world. People can become infected after breathing in *C. neoformans*, but usually people who have weakened immune systems are at most risk, particular those who have advanced HIV/AIDS [159]. Cryptococcosis usually affects the lungs or the central nervous system, however, sometimes life-threatening brain infections (cryptococcal meningitis) occur. In vitro co-culture experiments showed an ingestion and even replication of the yeast cells inside *A. castellanii* (enclosed in membrane-bound vesicles, the phagocytic vacuole) leading to the death of the amoeba [42]. Both passage in *Acanthamoeba castellanii* and *Dictyostelium discoideum* enhance virulence of *Cryptococcus neoformans* [42,160].

In addition to human pathogenic endocytobionts with public health importance, fungal parasites of FLA are also observed. *Cochlonema euryblastum* belongs to the order Zoopagales and specifically infects *Thecamoeba quadrilineata* [104]. After the uptake by this FLA conidia germinate to produce coiled thalli and within two days conidiogenous hyphae are produced which break through the protoplasmic pellicle of the amoeba. Some experts stated that fungal virulence may have evolved, and been maintained, as a countermeasure to predation by amoeba since pathogenicity in mammals is based on multiple virulence factors also required for fungal survival during interactions with non-vertebrate hosts [161].

## 7. Conclusions

There are numerous examples of such microorganisms which survive, grow, or proliferate inside amoebae [5]. The intracellular life in protozoa protects the endocytobionts from adverse environmental conditions whether of natural or artificial origin. In particular, cyst-forming FLA species and their endocytobionts are protected from biocides (as well as disinfectants, contact lens cleaners, other antiseptics, herbicides, pesticides, PCBs, and heavy metals) [121,162,163]. The fact that FLA can serve as hosts of and vehicles (vectors) for microorganisms, has an impact on the sector of public health. Not only is the resistance to biocides insides cysts increased but also virulence of some pathogens is boosted by exposure to FLA [73,134,135,136,137].

The host-endocytobiont relationships in FLA promote evolutionary processes and development of pathogenicity. It could mean that the amoebal passage is a prerequisite for the development of virulence factors—and, thus, for the development of human-pathogenic microorganisms. FLA then play an important role for their endocytobionts in the selection and evolution of virulence characteristics and the adaptation to (human) macrophages [5,36].

Since many of the pathogenic microorganisms which interact with FLA are water-borne there is a clear risk for quality in water networks. The resistance of FLA to drinking water treatments favors distribution of incorporated pathogens. Extracellular and intracellular biofilms possess an importance for evolutionary processes and the emergence of pathogens in biofilms. Therefore, in this regard, FLA can be viewed as evolutionary triggers or incubators.

## Figures and Tables

**Table 1:** Selection of microorganisms interacting with free-living amoebae (FLA), including pathogens with public health importance.

Species	Host Amoeba	References (Selection)
**BACTERIA**		
**In Situ Interaction**		
*Legionella*-like, *Legionella lytica* (*Sarcobium lyticum*)	*Acanthamoebae*, Naegleriae, Hartmanellae	[47,48,49,50]
*Legionella pneumophila*	Several FLA, e.g., *Acanthamoebae*	[51,52,53,54,55]
*Listeria monocytogenes*	*Acanthamoebae*	[56,57]
*Mycobacterium avium*	*Acanthamoebae*	[41,58]
*Afipia felis*	*Acanthamoebae*	[59]
*Pseudomonas saccharophilia*	*Acanthamoebae*	[60]
*Pseudomonas aeruginosa*	*Echinamoebae*; *Acanthamoebae*; *Hartmannellae*	[61,62]
*Ralstonia* (*Burkholderia*) *picketti*	*Acanthamoebae*	[62]
*Stenotrophomonas maltophilia*-Komplex	*Acanthamoebae*; *Naegleriae*	[63]
*Rickettsia*-like	*Acanthamoebae*	[39]
*Simkania nevegensis* (*Chlamydia*-like)	*Acanthamoebae*, *Naegleriae*, *Hartmannellae*; *Balamuthia mandrillaris*	[64,65,66]
*Neochlamydia hartmannellae*	*Hartmannellae*; *Hartmannella* (*Vermamoeba*) *vermiformis*	[67]
Candidatus *Mesochlamydia elodeae*	*Vannellae*, *Saccamoebae*	[68]
*Ehrlichia*-like	*Saccamoebae*	[61]
Candidatus *Paracaedibacter symbiosus*	*Acanthamoebae*	[69]
*Procabacter acanthamoebae* (Betaproteobacterium)	*Acanthamoebae*	[70]
*Amoebophilus asiaticus*	*Acanthamoebae*	[71]
**In Vitro Interaction**		
*Salmonella enterica*, *S. thyphimurium* (and other Salmonellae)	*Acanthamoebae*	[72,73]
*Yersinia enterocolitica*	*Acanthamoebae*	[74]
*Campylobacter jejuni*	*Acanthamoebae (Acanthamoeba polyphaga)*	[75]
*Arcobacter butzleri*	*Acanthamoebae (Acanthamoeba castellanii)*	[76,77,78]
*Mycobacterium* spp*.*, *M. leprae*	*Acanthamoebae*	[38,79,80]
*Staphylococcus aureus* (incl.MRSA)	*Acanthamoebae*	[81]
*Mobiluncus curtisii*	*Acanthamoebae*	[82]
*Mycobacterium leprae*	*Acanthamoebae*	[79]
*Mycobacterium xenopi*	*Acanthamoebae*	[83]
*Mycobacterium* spp; *Mycobacterium avium*	*Acanthamoebae*	[38,79,80]
Opportunistic Mycobacteria	*Acanthamoebae*	[41,80]
*Bacillus anthracis*	*Acanthamoeba castellanii Hartmannella* (*Vermamoeba*) *vermiformis*	[84]
*Acidovorax temperans*	*Naegleriae*	[85]
*Burkholderia cepacia* and *Burkholderia pickettii*	*Acanthamoebae*	[86]
*Caulobacter vibrioides*	*Echinamoebae*	[85]
*Flavobacterium johnsoniae*	*Naegleriae*	[85]
*Escherichia coli* O157	*Acanthamoebae*	[87]
*Helicobacter pylori*	*Acanthamoebae*	[88]
*Porphyromonas gingivalis*	*Acanthamoebae*	[89]
*Prevotella intermedia*	*Acanthamoebae*	[89]
*Chlamydophila pneumoniae*	*Acanthamoebae*	[90]
*Parachlamydia acanthamoebae*	*Acanthamoebae*	[91,92,93]
*Waddlia* sp., *Waddlia chondrophila*, other *Chlamydia*-like endocytobionts	*Acanthamoebae*, *Naegleriae*, *Hartmannellae*, *Hyperamoebae*, *Vahlkampfiae*, *Dictyostelium discoideum*	[85,94,95]
Other *Parachlamydia*-like endocytobionts	*Acanthamoebae*	[96,97]
*Coxiella burnetii*	*Acanthamoebae*	[98]
*Francisella tularensis*	*Acanthamoebae*	[99,100,101,102]
**PROTISTS AND FUNGI**		
**In Situ Interaction**		
*Paramicrosporidium* gen. nov	*Vannella* sp.	[103]
*Cochlonema euryblastum*	*Thecamoebae*; *Thecamoeba quadrilineata*	[104]
**In Vitro Interaction**		
*Cryptosporidium parvum*	*Acanthamoebae*	[105,106,107]
*Cryptococcus neoformans*	*Acanthamoebae*	[42]
**VIRUSES**		
**In Situ Interaction**		
*Adenoviridae*	*Acanthamoebae*	[108]
*Acanthamoeba polyphaga Mimivirus*	*Acanthamoebae*	[109]
*Pandoravirus inopinatum*	*Acanthamoebae*	[12,31]
*Pithovirus*	*Acanthamoebae*	[110]
**In Vitro Interaction**		
*Adenoviridae*	*Acanthamoebae*	[111]
Coxackie virus	*Acanthamoeba*	[112]
*Marseillevirus*	*Acanthamoebae*	[113]
*Megavirus chilensis*	*Acanthamoebae*	[114]
*Pandoravirus salinus*, *Pandoravirus dulcis*	*Acanthamoebae*	[115]
*Pithovirus sibericum*	*Acanthamoebae*	[116]
*Faustovirus*	*Vermamoeba vermiformis*	[46]
*Mollivirus sibericum*	*Acanthamoebae*	[117]
